# Minimizing Spatial Variability of Healthcare Spatial Accessibility—The Case of a Dengue Fever Outbreak

**DOI:** 10.3390/ijerph13121235

**Published:** 2016-12-13

**Authors:** Hone-Jay Chu, Bo-Cheng Lin, Ming-Run Yu, Ta-Chien Chan

**Affiliations:** 1 Department of Geomatics, National Cheng Kung University, Tainan City 700, Taiwan; honejay@mail.ncku.edu.tw (H.-J.C.); yudaniel178@yahoo.com.tw (M.-R.Y.); 2 Research Center for Humanities and Social Sciences, Academia Sinica, Taipei City 115, Taiwan; bclin@gate.sinica.edu.tw

**Keywords:** floating catchment area, particle swarm optimization

## Abstract

Outbreaks of infectious diseases or multi-casualty incidents have the potential to generate a large number of patients. It is a challenge for the healthcare system when demand for care suddenly surges. Traditionally, valuation of heath care spatial accessibility was based on static supply and demand information. In this study, we proposed an optimal model with the three-step floating catchment area (3SFCA) to account for the supply to minimize variability in spatial accessibility. We used empirical dengue fever outbreak data in Tainan City, Taiwan in 2015 to demonstrate the dynamic change in spatial accessibility based on the epidemic trend. The x and y coordinates of dengue-infected patients with precision loss were provided publicly by the Tainan City government, and were used as our model’s demand. The spatial accessibility of heath care during the dengue outbreak from August to October 2015 was analyzed spatially and temporally by producing accessibility maps, and conducting capacity change analysis. This study also utilized the particle swarm optimization (PSO) model to decrease the spatial variation in accessibility and shortage areas of healthcare resources as the epidemic went on. The proposed method in this study can help decision makers reallocate healthcare resources spatially when the ratios of demand and supply surge too quickly and form clusters in some locations.

## 1. Introduction

Dengue fever is the most serious arboviral disease, and has become a major health problem in most tropical countries in recent decades [1]. A recent estimate indicates that 390 million people annually have dengue infections, in which 96 million cases have clinical manifestations [2]. In Taiwan, the number of confirmed cases in 2015 reached a record high of 43,784 as reported by the Taiwan Centers for Disease Control (CDC) [3]. Among these cases, there were 22,777 cases in Tainan city, which is located in southern Taiwan. The demand for resources during the dengue outbreak in Tainan exceeded the capacity of individual health facilities. Thus, the management of hospital resources, including beds, equipment, and clinical manpower, and so on [4], in the existing hospital networks has become a significant issue [5].

For dengue infection, health facilities, such as clinics and hospitals, provide appropriate clinical management to avert patients’ severe outcomes and also provide either isolation wards or mosquito nets to reduce the chance of subsequent transmission [6]. Therefore, the effective use of health facilities will be critical for dengue prevention and treatment. To ensure adequate access to healthcare, policy makers of health departments need an accurate and reliable measure of accessibility so that areas with limited resources can be identified. Accessibility of healthcare can be classified according to two dimensions (potential versus revealed, and spatial versus aspatial) into four categories: potential spatial accessibility, potential aspatial accessibility, revealed spatial accessibility, and revealed aspatial accessibility [7].

Traditionally, hospital resources are modeled according to non-spatial factors, such as patient flow, patient needs, patient length of stay, and so on [4,8]. However, the pattern of locations of infection cases shows spatial clustering. To further improve the effective use of hospital resources from a spatial perspective, the potential spatial accessibility is considered in this research. Potential accessibility focuses on estimating the probability of entering the healthcare system, while spatial accessibility emphasizes the importance of the fact that spatial distance can influence the probability of entering the healthcare system.

Spatial accessibility is determined by the spatial distribution between supply and demand. The two-step floating catchment area (2SFCA) is a popular method [9] for measuring the spatial accessibility of health facilities [10,11,12]. It is a two-step process to compute the supply-to-demand ratio within a catchment area around each demand location. To improve the equal access within a catchment in the 2SFCA, a stepwise decaying of accessibility within each catchment is introduced in the enhanced 2SFCA (E2SFCA) method [13]. Furthermore, the stepwise decay is improved to a continuous function by involving a kernel density [14] or a Gaussian function [15]. However, the limitation of the 2SFCA is the overestimation effect when there are more supply locations within a catchment area. The three-step floating catchment area (3SFCA) method is proposed to introduce competition among supply locations. It assumes that probable demand is influenced by the availability of other nearby supply locations [16].

Many simulation methods and tools for hospital resources management are evaluated at the level of the individual health facility [8,9,10,11]. However, a challenge may be posed to the provision of healthcare when there is a sudden surge in demand, such as in the case of a multi-casualty incident or the outbreak of an infectious disease. The integration and allocation of hospitals’ resources in a region is thus important. In this study, the modeling of resource management among the health facilities network is conducted in order to find out the bed requirement of each hospital during an epidemic event. This work aims to develop a dynamic spatial accessibility model through the simulation of the healthcare resources. For the optimal spatial accessibility distribution, this study integrates particle swarm optimization (PSO) and 3SFCA to minimize the spatial variability of accessibility for preventing the shortage problem in local areas.

## 2. Materials and Methods

### 2.1. 3SFCA

The 2SFCA method evaluates the spatial access to healthcare in two steps. First, for each supply location *j*, it searches for all demand locations *k* that are within a threshold travel distance (*d*_0_). The supply-to-demand ratio for a service site ($R_{j}$) is calculated as:

(1) $$R_{j}=\frac{S_{j}}{\sum_{k\in \{d_{kj}\leq d_{0}\}\;}D_{k}}$$

where *S_j_* is the supply at service site *j* and $D_{k}$ is the patient size/demand at location *k* which is within the catchment of service site *j*. The threshold distance (*d*_0_) is used to define the catchment size. *d*_0_ within the catchment area are 4.5 km and 3.5 km for academic and regional hospitals. This is determined based on coverage of over 90% of patient points during the outbreak period.

The accessibility of a location is the summation of the supply-to-demand ratios of all the service sites within the catchment. For each demand location *i*, search all supply locations *j* that are within a threshold travel distance (*d*_0_) in location *i*. *A_i_* is the spatial accessibility at location *i*:

(2) $$A_{i}=\underset{j\in \{d_{ij}\leq d_{0}\}}{\sum }R_{j}$$

In this study, the supply is the number of beds and the demand is the number of patients with dengue infection. In the 3SFCA, this equation integrates the capacity and distance effect with the FCA method to articulate patient selection of services [17].

(3) $$R_{j}=\frac{S_{j}}{\sum_{k\in \{d_{kj}\leq d_{0}\}}Prob_{kj}D_{k}W_{kj}}$$

(4) $$Prob_{kj}=\frac{C_{j}W_{kj}}{\sum_{j\in \{d_{kj}\leq d_{0}\}}C_{j}W_{kj}}$$

where *Prob_kj_* is the probability of patient at location *k* visiting service site *j*. *W_kj_* is the inverse distance weight between patient’s location *k* and service site *j*. The accessibility of a location ($A_{i}$) is the summation of the supply-to-demand ratios considering the capacity effect.

(5) $$A_{i}=\underset{j\in \{d_{ij}\leq d_{0}\}}{\sum }Prob_{ij}R_{j}W_{ij}$$

(6) $$Prob_{ij}=\frac{C_{j}W_{ij}}{\sum_{j\in \{d_{ij}\leq d_{0}\}}C_{j}W_{ij}}$$

where *Prob_ij_* is the probability of service site *j* in catchment location *i*; $C_{j}$ is the capacity of service site *j*. $W_{ij}$ is the inverse distance weight between catchment location *i* and service site *j*.

The $W_{ij}\;$is calculated from a Gaussian function, which means that the access to a physician diminishes with distance. The function is calculated as follows [18]:

(7) $$W_{ij}=e^{-dij^{2}/\beta }$$

*d_ij_* is the travel distance between patient or catchment *i* and hospital *j*, while β is the weighting parameter.

In this study, spatial accessibility with the period *t*, $A_{i}\left(t\right)$ can be defined as the following.

(8) $$R_{j}\left(t\right)=\frac{S_{j}\left(t\right)}{\sum_{k\in \{d_{kj}\leq d_{0}\}}Prob_{kj}D_{k}\left(t\right)W_{kj}}$$

(9) $$A_{i}\left(t\right)=\underset{j\in \{d_{ij}\leq d_{0}\}}{\sum }Prob_{ij}R_{j}\left(t\right)W_{ij}$$

### 2.2. Optimal Resource Allocation

To decrease the spatial variation in accessibility, the objective function is to minimize the standard deviation (SD) and maximize the average (AV) of the spatial accessibility distribution during the periods. The decision variables are the supply in hospitals’ resources (bed capacity for each hospital), but they are constrained by upper and lower limits,$\;S_{u}$ and $S_{l}$, and are summed to obtain the original supply.

(10) $$min\;\underset{t}{\sum }SD\left(A\left(t\right)\right)$$

(11) $$max\;\underset{t}{\sum }AV\left(A\left(t\right)\right)$$

(12) $$s.t.\;S_{l}\left(t\right)\leq S\left(t\right)\leq S_{u}\left(t\right)$$

(13) $$\underset{t}{\sum }S\left(t\right)=\underset{t}{\sum }S_{0}\left(t\right)$$

Wang and Tang (2011) [19] used quadratic programming to minimize the variance of accessibility by readjusting the amounts of service supplies. In this study, the optimization search method is the PSO, which is a derivative-free global optimization technique developed by Kennedy and Eberhart [20]. The PSO is inspired by the social behavior of bird flocking or fish schooling. The PSO provides a population-based search procedure in which individuals called particles change their position with time. In this study, 100 particles were used. In the PSO, each particle adjusts its position according to its own experience, and according to the experience of a neighboring particle, making use of the best position encountered by itself and its neighbor.

### 2.3. Data and Material

**Figure 1:**
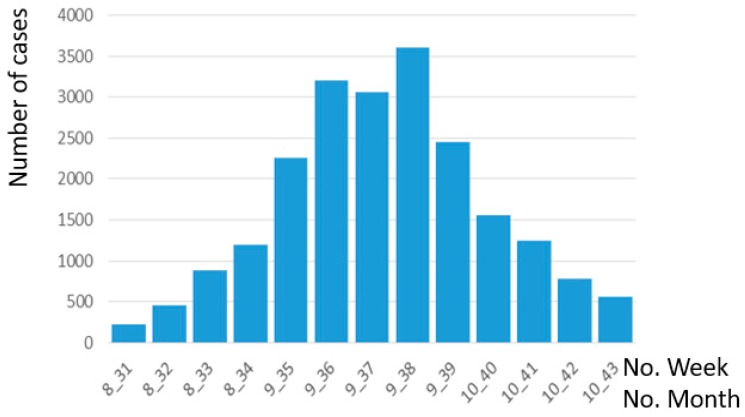
Number of cases in dengue outbreak from week 31 to week 43.

**Figure 2:**
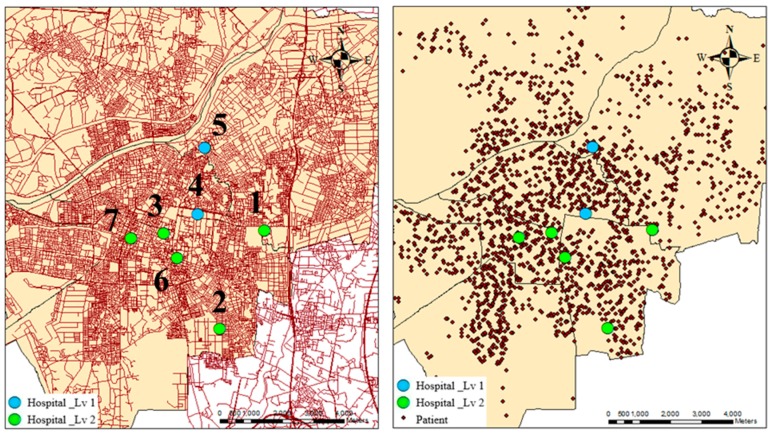
(**left**) Hospital network (level 1 (hospitals #4 and #5) indicates academic hospitals, and level 2 (hospitals #1, #2, #3, #6, and #7) means regional hospitals) and (**right**) patient patterns in Tainan City.

Empirical dengue fever outbreak data for Tainan City, Taiwan in 2015 were publicly downloaded from Department of Health, Tainan City Government [3]. The numbers of patients with dengue infections from week 36 to week 39 in 2015, referred to as weeks 1 to 4 in the result section, are shown in Figure 1; the number of cases is over 2400 per week. This study considers the data for these four peak weeks as a case study. The demand is the number of patients with dengue infection. Seven hospitals provided the supply. The hospitals and health network in Tainan City and the location of patient cases during the four weeks are shown in Figure 2. The health facilities in Taiwan are classified into three levels: level 3 is physician clinics, level 2 means regional hospitals, and level 1 indicates academic hospitals. In Figure 2, two hospitals are at level 1 (hospitals #4 and #5) but five hospitals are at level 2 (hospitals #1, #2, #3, #6, and #7). Based on hospital vacancy rates, the original supply in seven hospitals is 74, 79, 160, 106, 65, 87, and 152 beds per week. Before the modeling, the regional supply and demand ratios are 0.26, 0.27, 0.23, and 0.38 during the four weeks.

## 3. Results

**Figure 3:**
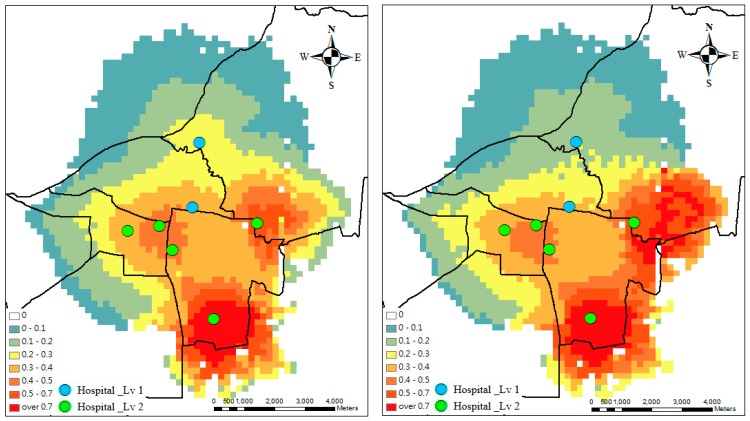
Accessibility without (**left**) and with (**right**) capacity at week 1.

Generally, highest accessibility is found in urban areas with service sites, while suburban areas have low accessibility. Figure 3 shows the spatial accessibility in terms of supply-to-demand ratio without and with considering the capacity. The accessibility value is high in the north without the capacity effect. After considering the capacity effect, the spatial accessibility decreases during the outbreak period near the academic hospital that is in the northern part, but increases near a regional hospital in the eastern part. Therefore, the spatial variability in accessibility increases after considering the capacity effect.

**Figure 4:**
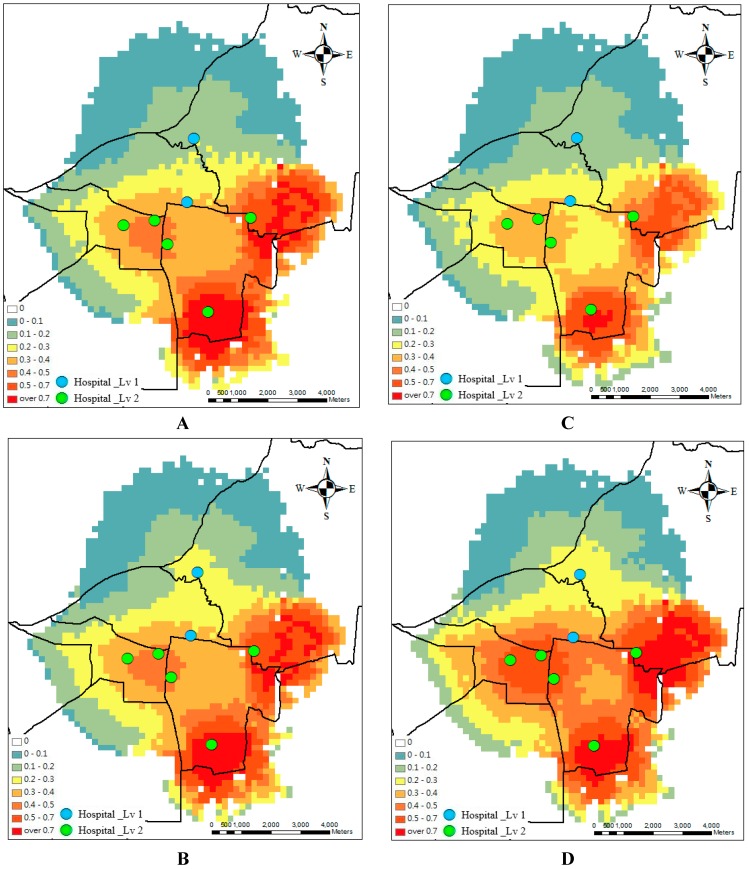
Accessibility before the optimal design at week 1 (**A**); week 2 (**B**); week 3 (**C**) and week 4 (**D**).

**Figure 5:**
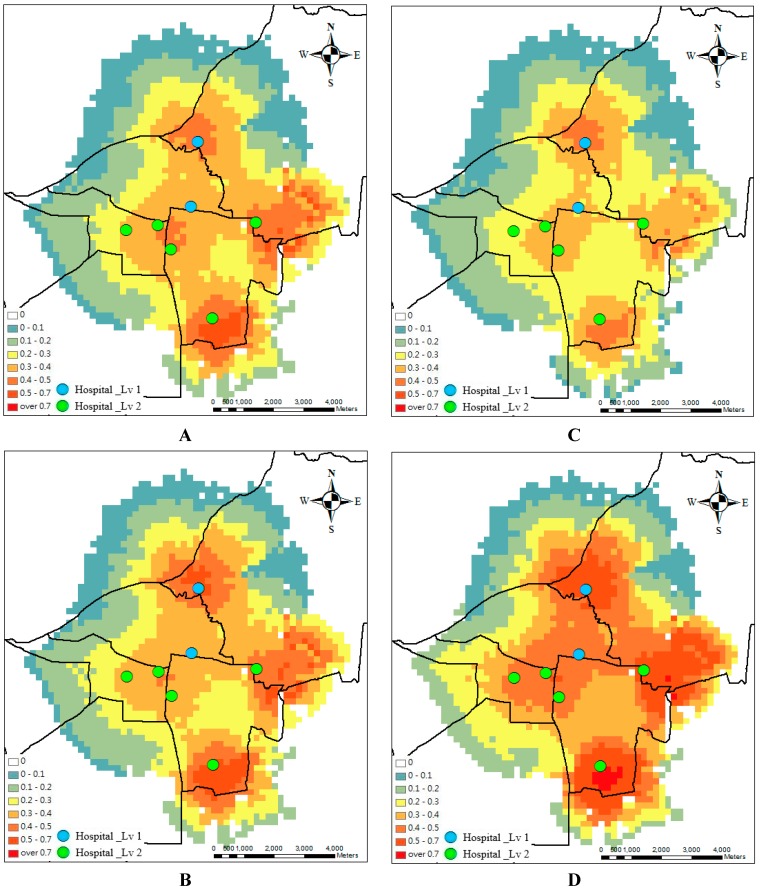
Accessibility after the optimal design at week 1 (**A**); week 2 (**B**); week 3 (**C**) and week 4 (**D**).

Optimal design for access relies upon understanding the hospitals and epidemic processes involved. To minimize spatial variability in accessibility, the optimal model is applied. Figure 4 and Figure 5 show the spatial accessibility before and after the optimal design during the four weeks. Originally, the spatial accessibility concentrates in the southern and eastern parts but is scarce in the northern part. After the optimal design, the area of the scarce accessibility is reduced and the uniform spatial accessibility distribution is determined. The hotspots of the accessibility are around the hospital clusters.

In Table 1, the average SD of spatial accessibility during the four weeks is 0.20 before optimal design, but the average SD decreases to 0.13 after the optimal design. The variation in the accessibility can be improved after the optimal design. The results also show that the spatial accessibility is sufficient near the hospitals, but the boundary area is not enough. Prior to the optimal design, 45% of areas have lower potential spatial accessibility of health services than 0.2, but only 39% of the areas are lower than 0.2 after the optimal design. The area with low accessibility can be reduced after the optimal design. Table 2 shows the original and optimal supply (bed capacity) of each hospital after the design. The results show that the optimal supply in two big hospitals should be increased, but the optimal supply of small ones should be decreased.

## 4. Discussion

Spatial accessibility is recognized as important information for the management of regional hospital resources [9]. The disparity in spatial distribution of health facilities can be evaluated and considered in the process of resource allocation [14]. In this study, the 3SFCA method was adopted to measure spatial accessibility. Theoretically, the spatial accessibility in 3SFCA includes regional availability and regional accessibility [21]. The regional availability focuses on the relationship between supply (hospital resources) and demand (population or patients) in a specific area. However, regional accessibility focuses on the spatial resistance, such as the distance between a hospital and patients. The 3SFCA method can integrate these two aspects, considering the complex interaction among the supply, demand, and the location of hospitals and patients in different regions to estimate the spatial accessibility of healthcare in a definite area.

The dengue epidemic varied over time so that a dynamic model is needed. This study demonstrated the dynamic 3SFCA method for healthcare resources. In the 3SFCA, the three main factors, the hospital catchment area, the hospital capacity, and the weighting parameter in a decay function, have to be identified. These parameters are always experimental values, depending on the regional conditions in previous studies [13,16,18]. It is difficult to perform evaluations in a dynamic situation, such as an outbreak of dengue infection. To determine the optimal parameters, the supervised method is adapted to evaluate the appropriate value according to the weekly number of dengue cases. The evaluation of spatial accessibility can be adapted to the region during the period by applying these optimal parameters.

World Health Organization (WHO) guidelines on clinical management of dengue fever [22] classify dengue infected patients into three groups, including Group A (to be sent home), Group B (to be referred for in-hospital management), and Group C (requiring emergency treatment and urgent referral). Therefore, only Group B and Group C will need hospital resources. In this study, we focused on moderate to severe cases which need hospitalization. During the studied period, Tainan City suffered the worst dengue outbreak. One of the academia hospitals in Tainan City reported that 4787 patients needed intensive care unit (ICU) beds from 31 July to 31 November 2015 [23]. This is a very high demand for the hospital. From the viewpoint of hospital management and the health insurance system in Taiwan, the number of doctors will be determined by the number of beds or patients you have. Thus, the most direct indicator for measuring the supply is the capacity of the beds in each hospital. In general, the hospitals will not use all this capacity during their daily operation. Once an emergency situation arises, they may call for doctors and nurses from other hospitals or retired volunteers to help them care for the extra patients. However, the ceiling of the serving capacity is that maximum constraint number.

Considering hospitals’ capacity and accessibility, which is the ratio of supply to demand, accessibility decreases because demand increases dramatically in academic hospitals. The supply capacity of a service site affects patients’ selection. Patients seek more services in the big hospitals. The large supply capacity increases probability for attracting large numbers of patients. Therefore, Figure 3 shows that the accessibility in the northern part decreases, considering the hospitals’ capacity. After the optimal design (compare Figure 4 and Figure 5), the medical referral system should play an active role in adjusting the supply of hospitals, such as beds and doctors. Under this design, there will be more balanced accessibility among all hospitals. The accessibility in the northern academic hospitals can be improved.

In fact, people do not necessarily go to the closest facility. Because Taiwan has 99% national health insurance coverage, everyone can choose the hospital they would like to go to. The price is different when you go to different level hospitals, but the same within the same level hospitals. Patients in different age groups or with different social economic status (SES) might have different medical seeking behaviors. However, we did not have detailed demographic information here. Thus, we cannot explore these patterns. Another study found that patients are willing to travel farther to distant medical facilities to get treatment [24]. However, in our study, we think those demographic effects might be more obvious for mild dengue cases than for moderate to severe cases. Because we do not have a good referral system [25], patients will normally choose the nearest hospital for emergency care or intensive care. The type of healthcare system is not the major concern for the patients because they charge the same price if they are at the same level.

To determine the capacity of the hospital resources is difficult, because of the complex relationship between resources, utilization, and patient flow of different groups of patients. These complex factors are considered in many models to evaluate the capacity, such as discrete event simulation [26,27]. Furthermore, the outbreak of infectious disease or a natural disaster is considered when designing the surge capacity [28,29]. However, the hospital resources in these outbreaks cannot be evaluated only based on a large number of patient admissions. The spatial distribution of the patients in the region is also an important factor. In the spatial domain, the immediate measuring of spatial accessibility and the optimal analysis are more effective for allocation of hospital resources.

The outbreak of infectious disease has the potential to generate a large number of patients in an area [30,31]. The increase in patients will lead to overcrowding in an individual hospital during the period of the outbreak, such that the quality of care will be reduced [32]. For the treatment of mild dengue, temporary facilities for early screening or diagnosis are possible. However, moderate to severe dengue cases (such as those which are the focus of this study) need more intensive care and support, in which case temporary facilities might not be a good solution. Therefore, the management of resources in a regional hospital network becomes more and more critical for hospitals [5], such as the Regional Emergency Operation Center (EOC) in Tainan, which contains nine regional hospitals [33]. To detect the uniform distribution of spatial accessibility, the PSO is used to find the optimal hospital resources from each solution of hospital resources in the network. Although the overall resources do not experience an increase, the resources could be allocated in such a manner as to decrease the variation in spatial accessibility. After the optimal design, the areas of low accessibility might decrease dramatically, with a more balanced relationship between supply and demand in the whole Tainan city area. In fact, the reallocation of beds among different hospitals is not feasible in the real world. Every hospital has its maximum capacity due to limitations of both space and medical staff. Therefore, we considered each hospital’s number of beds as their supply constraint. If information on the number of specialized medical staff can be known, the model can also consider multiple variables to represent supply.

### Limitations

This study had some limitations. First, we did not consider the surge capacity of each hospital because of limited availability of the data, so the bed requirements of a hospital could not be increased. Second, the dengue fever cases and the static bed occupancy rate of each hospital do not correspond to the same period. The ideal situation is to integrate the daily occupancy rate to dynamically re-evaluate the spatial accessibility every day. Third, we did not have the number of dengue infections with different severity every week. Thus, we could only use the total number of dengue infections as our demand, which might over-estimate our demand. However, it can also be treated as the worst scenario of the dengue outbreak.

## 5. Conclusions

The purpose of this study was to understand how to minimize variability among hospitals in terms of their accessibility. The spatial and temporal patterns of healthcare accessibility were evaluated and the impact of healthcare resources was simulated during a dengue outbreak. The capacity effect based on the fact that people do not necessarily go to the closest facility was also considered. The PSO model was used to decrease the spatial variation in accessibility and areas with shortage of healthcare resources by readjusting the amounts of service supplies. In this case, the distribution of spatial accessibility in the Tainan city area was non-uniform because the relationship between supply and demand is unbalanced in the north boundary area. To decrease the spatial variation in accessibility and areas with healthcare resource shortages, PSO model was used to readjust the amounts of service supplies. The optimal distribution of spatial accessibility can be estimated by the optimal allocation of hospital resources, so that policymakers can implement appropriate healthcare policies to cope with serious epidemics, raise the quality of healthcare, and decrease casualties.

## Figures and Tables

**Table 1:** Regional standard deviation (SD) of accessibility before and after the optimal design.

Week	Accessibility SD before Design	Accessibility SD after Design	Differences
1	0.22	0.13	−0.09
2	0.21	0.13	−0.08
3	0.16	0.11	−0.05
4	0.22	0.16	−0.06
Average	0.20	0.13	−0.07

**Table 2:** Supply before and after the optimal design.

No.	Hospital Level	Original Supply	Optimal Supply at Week 1	Optimal Supply at Week 2	Optimal Supply at Week 3	Optimal Supply at Week 4
1	2	74	45.5	48.8	48.8	51.6
2	2	79	44.5	49.4	48.4	66.2
3	2	160	90.0	126.0	73.9	111.9
4	1	106	148.5	136.4	141.8	142.2
5	1	65	160.0	159.5	160.0	160.0
6	2	87	104.5	73.1	121.5	61.0
7	2	152	130.0	129.8	128.6	130.0
